# *γ*-Tocotrienol suppresses prostate cancer cell proliferation and invasion through multiple-signalling pathways

**DOI:** 10.1038/sj.bjc.6604763

**Published:** 2008-11-11

**Authors:** W N Yap, P N Chang, H Y Han, D T W Lee, M T Ling, Y C Wong, Y L Yap

**Affiliations:** 1 Davos Life Science Pte. Ltd., Cancer Research Laboratory, 11 Biopolis way, #07-03, The Helios 138667, Singapore; 2 Department of Anatomy, Cancer Biology Lab, Li Ka Shing Faculty of Medicine, The University of Hong Kong (HKU), 1/F, Laboratory Block, 21 Sassoon Road, Hong Kong SAR

**Keywords:** tocotrienol, prostate cancer, Id-1, E-cadherin, Docetaxel, chemosensitising

## Abstract

Tocotrienol-rich fraction (TRF) has demonstrated antiproliferative effect on prostate cancer (PCa) cells. To elucidate this anticancer property in PCa cells, this study aimed, first, to identify the most potent isomer for eliminating PCa cells; and second, to decipher the molecular pathway responsible for its activity. Results showed that the inhibitory effect of *γ*-tocotrienol was most potent, which resulted in induction of apoptosis as evidenced by activation of pro-caspases and the presence of sub-G_1_ cell population. Examination of the pro-survival genes revealed that the *γ*-tocotrienol-induced cell death was associated with suppression of NF-*κ*B, EGF-R and Id family proteins (Id1 and Id3). Meanwhile, *γ*-tocotrienol treatment also resulted in the induction of JNK-signalling pathway and inhibition of JNK activity by a specific inhibitor (SP600125) was able to partially block the effect of *γ*-tocotrienol. Interestingly, *γ*-tocotrienol treatment led to suppression of mesenchymal markers and the restoration of E-cadherin and *γ*-catenin expression, which was associated with suppression of cell invasion capability. Furthermore, a synergistic effect was observed when cells were co-treated with *γ*-tocotrienol and Docetaxel. Our results suggested that the antiproliferative effect of *γ*-tocotrienol act through multiple-signalling pathways, and demonstrated for the first time the anti-invasion and chemosensitisation effect of *γ*-tocotrienol against PCa cells.

Prostate cancer (PCa) is the most common type of cancer in developed countries. It is responsible for more male deaths than any other cancers, except for lung and bronchial cancer (Ries *et al*, 2006). Most PCas present themselves as mixtures of androgen-dependent and androgen-independent cells during clinical diagnosis. They initially respond to androgen ablation therapy by undergoing programmed cell death (apoptosis) (Craft *et al*, 1999). However, patients with advanced PCa develop hormonal refractory disease that results in a fatal effect because of the growth of androgen-independent PCa cells (Feldman and Feldman, 2001). Until recently, the only chemotherapeutic drug that shows improvement in survival of patients, Docetaxel, can only extend the overall survival by 2 1/2 months (Chowdhury *et al*, 2007). Furthermore, the treatment is associated with significant side effects. Therefore, an alternative methodology to enhance the apoptotic response is necessary to develop new therapeutic drugs for the treatment of PCa.

Natural products have historically been a rich source of biologically active compounds for drug discovery (Schiff *et al*, 1979). Tocotrienols (T3) are important plant vitamin-E constituents found in palm oil. Together with tocopherols (T), they provide a significant source of antioxidant activity to all living cells (Ahmad *et al*, 2005; Mazlan *et al*, 2006). This common antioxidant attribute reflects the similarity in chemical structures of the tocotrienols and the tocopherols, which differ only in their structural side chain (contains farnesyl for tocotrienol or saturated phytyl side chain for tocopherol). The common hydrogen atom from the hydroxyl group on the chromanol ring acts to scavenge the chain-propagating peroxyl-free radicals. Depending on the locations of methyl groups on their chromanol ring, tocopherols and tocotrienols can be distinguished into four isomeric forms: alpha (*α*), beta (*β*), gamma (*γ*), and delta (*δ*).

Apart from tocotrienol's antioxidant, anti-inflammatory, antiangiogenic, antineurodegeneration, antihypercholesterolemic and antimicrobial properties, emerging *in vitro* and *in vivo* evidences have manifested the anticancer activity of tocotrienol on numerous human cancer cells including prostate, breast, colon, liver and gastric (Agarwal *et al*, 2004; Nesaretnam *et al*, 2004; Sylvester and Shah, 2005; Eitsuka *et al*, 2006; Kumar *et al*, 2006; McAnally *et al*, 2007) (reviewed in (Sen *et al*, 2007)). For example, a recent study (Srivastava and Gupta, 2006) has demonstrated that treatment of PCa cell lines (LNCaP, DU145, PC-3) with tocotrienol-rich fraction (TRF) resulted in significant decreases in cell viability and colony formation capability. More interestingly, TRF was able to selectively spare the normal human prostate epithelial cells, an important characteristic of effective chemoprevention strategy. However, it should be noted here that the TRF used in the study was a mixture of various vitamin-E isomers. Thus, it was not possible to determine which isomer possesses the highest potency in eliminating proliferating prostate cancer cells selectively, let alone the discovery of molecular pathways responsible for the inhibitory effect. In other studies (McAnally *et al*, 2003; Conte *et al*, 2004; Vraka *et al*, 2006) *γ*-T3 isomer was reported to suppress prostate cancer cell growth with IC_50_ values for PC-3 between 25 and 51 *μ*M, although how the cell responds to the other isomers was not investigated.

Although scientific evidences evoking suppression of cancer cells by T3 are increasing, the exact pathways involved in T3-induced apoptosis remain elusive. Initially, T3 treatment was shown to induce apoptosis through a p53-dependent mechanism (Agarwal *et al*, 2004). Using p53^+^ human colon carcinoma RKO cells, researchers demonstrated that TRF-induced activation of the Bax gene through upregulation of the p53 protein. This was associated with the release of cytochrome *c* from nucleus to cytosol, induction of Apaf1 oligomerisation as well as activation of caspase 9. Meanwhile, the T3-induced p53 expression was also found to result in downregulation of Bcl-2 level, which eventually triggers apoptotic-signalling cascade by increasing the bax/bcl-2 ratio. In addition to p53 pathway, phosphatidylinositol-3-kinase-dependent kinase (PI3K)/PI3K-dependent (PDK1) mitogenic-signalling cascade (Samant and Sylvester, 2006) and NF-*κ*B (Ahn *et al*, 2007) pathways have both been suggested to contribute to T3-induced apoptosis. For example, in *γ*-T3-treated cancer cells, inactivation of PDK1 and Akt as well as downregulation of their downstream effectors such as FLICE-inhibitory protein (FLIP) and phospho-NF-*κ*B were observed. FLIP reduction was shown to promote the cleavage of caspases 3 and 8, which results in growth arrest and apoptosis in T3-treated cells. Taken together, it seems that T3-induced apoptosis in cancer cells may involve activation/inactivation of multiple-signalling pathways.

## Materials and methods

### Prostate cancer cell lines, cell culture conditions and chemicals

The human androgen-dependent PCa cells (LNCaP), human androgen-independent PCa cells (PC-3) (ATCC, Rockville, MD, USA) were maintained in their respective medium recommended by ATCC (Invitrogen, Carlsbad, CA, USA) supplemented with 2 mmol l^−1^ L-glutamine, 10% fetal calf serum (FCS) and 2% penicillin streptomycin at 37°C in 5% CO_2_. The immortalised human prostate epithelial cells (PZ-HPV-7) (ATCC, Rockville, MD, USA) were maintained in keratinocyte serum-free medium (K-SFM) supplemented with bovine pituitary extract (BPE, 0.05 mg ml^−1^) and human recombinant epidermal growth factor (EGF, 5 ng ml^−1^). Docetaxel (Calbiochem, San Diego, CA, USA) and JNK inhibitor, SP600125 (Sigma-Aldrich, St Louis, MO, USA), were dissolved in dimethylsulphoxide (DMSO). The treatment solutions were diluted in culture medium to obtain the desired concentrations.

### Tocotrienol and tocopherol isomers

Tocotrienol and tocopherol isomers were extracted and purified from palm oil using Davos separation technology. The extraction facility is located at Tuas Singapore. Crude palm oil feed was purchased from Kuala Lumpur Kepong Berhad. Using the corresponding tocotrienol isomers as the reference standard, the purity of T3 and T isomers was verified to be ⩾97% by high performance liquid chromatography percentage area (% area).

### Cell viability study and time course experiment

For cell viability study, 5 × 10^3^ cells resuspended in 100 *μ*l medium were plated into each well of a 96-well plate. The cells were then treated with different concentrations (20, 40, 60, 80, 100 *μ*M) of the vitamin-E isomers for 24- and 48-h. After the treatment, 20 *μ*l of MTT solution was added into each well and the cells were incubated at 37°C for 2 h. The formazan crystals were then resuspended in 200 *μ*l of DMSO and the intensity at 595 nm were measured. For JNK inhibitor study, cells were pre-treated with 20 *μ*M of SP600125 for 8 h prior to the addition of vitamin-E isomers. For time course study, 5 × 10^3^ cells (LNCaP and PC3) were treated with IC_50_ concentrations of the vitamin-E isomers and were subjected to the MTT assay at the indicated time point. If IC_50_ for the isomer is >100 *μ*M, 100 *μ*M will be used as treatment dosage. Each experiment was repeated three times in triplicate wells and the growth curves showed the means and s.d.

To test the effect of *γ*-T3 on the cytotoxicity of Docetaxel, cells were pre-incubated with *γ*-T3 for 3 h before addition of 20 and 100 nM of Docetaxel. After 24 h, cells were subjected to western blotting and MTT assays respectively.

### Flow cytometry

Cell cycle distribution was examined using flow cytometry. Briefly, cells were harvested by trypsinisation, fixed in 70% ethanol at 4°C overnight, and then resuspended in PBS. After incubation at 4°C overnight, 2 × 10^6^ cells were incubated with 20 *μ*g ml^−1^ propidium iodide and 2 mg RNase A for 15 min at 37°C. Cells were then examined by BD SLRII cytometer and the results were analysed using ModFit software (Becton Dickinson, Mountain View, CA, USA). Data were expressed as the percentage of cell cycle distribution in the entire population.

### Matrigel-invasion assay

Matrigel-invasion assay was performed according to a method published earlier with modifications (Albini *et al*, 1987). Briefly, the invasive androgen-independent PCa cells (PC-3) were pre-incubated in a serum-free RPMI-1640 medium with or without *γ*-T3 isomers for 24 h. The PC-3 cells, (2.5 × 10^5^) resuspended in 500 *μ*l of serum-free RPMI-1640 containing 0.1% bovine serum albumin, were then added to the upper chamber of an 8-*μ*m pore size insert (Millipore, Bedford, MA, USA) manually coated with Matrigel (0.5 mg ml^−1^) (BD Bioscience, Bedford, MA, USA). Five hundred microliters of invasion buffer containing fibronectin (10 *μ*g ml^−1^) and RPMI-1640 supplemented with 10% FCS were added in the lower chamber as a chemo-attractant. The PC-3 cells were incubated at 37°C for 24 h in 5% CO_2_-humidified conditions. At the end of incubation period, inserts were stained with Diff-Quick staining solution (Fischer Scientific). Non-invaded PC-3 cells on the inside of the insert were scraped off with a cotton swab. The PC-3 cell invasions were then examined by a phase-contrast microscope. The invaded cells were extracted using extraction buffer (Millipore, Bedford, MA, USA) and the cell number was estimated based on absorbance at 595 nm.

### Western blotting

Detailed protocols have been described earlier (Chu *et al*, 2006). Briefly, cell lysates were prepared by suspending cell pellets in lysis buffer (50 mmol l^−1^ Tris-HCl (pH 8.0), 150 mmol l^−1^ NaCl, 1% NP40, 0.5% deoxycholate, 0.1% SDS, 1 mg ml^−1^ aprotinin, 1 *μ*g ml^−1^ leupeptin and 1 mmol l^−1^ phenylmethylsulphonyl fluoride). For nuclear protein extraction, NucBuster™ protein extraction kit was used. Protein concentration was measured using the DC Protein Assay kit (Bio-Rad, Hercules, CA, USA). Equal amount of protein (30 *μ*g) was loaded onto a 10% SDS polyacrylamide gel for electrophoresis and then transferred onto a polyvinylidene difluoride membrane (Amersham, Piscataway, NJ, USA). The membrane was then incubated with primary antibodies for 1 h at room temperature against E-cadherin (BD Biosciences, Bedford, MA, USA), *α*-catenin, *β*-catenin, *γ*-catenin, Id-1, Id-3, EGF-R, phosphor-c-jun, phospho-ATF2, cleaved PARP, vimentin, *α*-smooth muscle actin, twist (Santa Cruz Biotechnology, CA, USA), Phospho-i*κ*B-*α* (Ser32/36), Phospho-IKK *α* (Ser180)/IKK *β* (Ser181), Phospho-SAPK/JNK (Thr183/Tyr185) G9, SAPK/JNK, NF-*κ*B p65 (5A5) (Cell Signaling Technology Inc., Beverly, MA, USA), Snail (Abcam, Cambridge, UK). After incubation with appropriate secondary antibodies, signals were visualised by ECL western blotting system (Amersham, Piscataway, NJ, USA). Expression of *β*-actin and histone H1 were assessed as an internal loading control for total cell lysate and nuclear protein lysate, respectively.

## Results

### Antiproliferation effect of vitamin-E isomers

PCa cells were treated with vitamin-E isomers for 24- and 48-h at increasing dosage (low: 20 *μ*M, medium: 40 *μ*M and high: 80 *μ*M) and for varying time points. Our results showed that vitamin-E isomers did not affect significantly the proliferation rate of normal prostate epithelial cells (PZ-HPV-7), but significantly suppressed the proliferation of LNCaP and PC-3 (Table 1A). The dose to suppress 50% cell growth (IC_50_) in LNCaP and PC-3 was inversely proportional to the length of incubation time. Surprisingly, PC-3 cells were more sensitive to the growth inhibition of the vitamin-E isomers than LNCaP cells. The inhibition of cell proliferation was significantly stronger for T3 isomers in PC-3, particularly for *γ*-T3, which showed a dose- and time-dependent inhibition (Table 1B). Although *δ*-T3 was more potent in suppressing cell growth in LNCaP (Table 1A), the IC_50_ value was significantly higher than that for *γ*-T3 in PC-3. Separately, *γ*-T was also found to induce apoptosis in LNCaP cells (Jiang *et al*, 2004) at a dose similar to *γ*-T3. Based on the IC_50_ values in PC-3 cells incubated with various isomers for 24-h, the order of inhibitory effect is *γ*-T3>*δ*-T3>*β*-T3>*γ*-T>*δ*-T≈*α*-T3≈*α*-T≈*β*-T. For the subsequent experiments, we investigated the most potent isomer for PC-3 (*γ*-T3) as they are in general considered more invasive and resistant to chemotherapeutic agents compared with LNCaP cells (Coppe *et al*, 2004; Ghosh *et al*, 2005).

**Figure 1:**
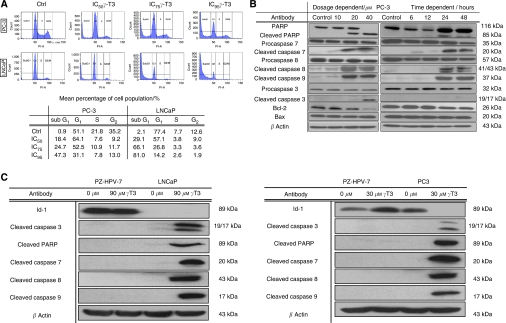
Induction of apoptosis by *γ*-T3 treatment. (**A**) Cell cycle analysis by flow cytometry. Control cells and treated cells incubated with *γ*-T3 at IC_50_ for 24-h were subjected to flow cytometry analysis. Note that the sub-G1 population appears after treatment. (**B**) IC_50_ time-dependent and 24-h dose-dependent activation (in hrs and *μ*M respectively) of the pro-apoptosis pathway in PC-3. Note that *γ*-T3 induces activation of the critical molecules (cleaved caspase 3, 7, 8, 9, PARP) and modulate the ratio between the amounts of bcl-2 and bax in a cell dose- and time-dependent fashion. (**C**) IC_50_ *γ*-T3 activates pro-apoptotic genes and suppresses pro-survival genes expression on LNCaP and PC-3 but not on non-tumorigenic prostate epithelial cells (PZ-HPV) for 24-h incubation period.

To study the mechanism responsible for *γ*-T3-induced growth inhibition, cell cycle distribution of the cells with or without *γ*-T3 treatment for 24 h were analysed by flow cytometry. Consequently, treatment of cells with *γ*-T3 (IC_50–95_) resulted in an induction of sub-G_1_ cell population, indicating the presence of apoptotic cells after the treatment (Figure 1A). The proportion of apoptotic cells (sub-G_1_ fraction) increased in a dose-dependent manner. It is noteworthy that although *γ*-T3 was previously reported to induce G_1_ arrest in some cell lines (Mo and Elson, 1999), we did not observe a significant increase of G_1_ population in prostate cancer cells that were treated with *γ*-T3. Consistent with the induction of sub-G_1_ cell population in flow cytometry, activation of procaspase 3, 7, 8, 9 as well as PARP, as evidenced by the appearance of the cleaved products, were observed in PC-3 cells treated with different *γ*-T3 dosage for 24 h. Downregulation of bcl-2 was also detected after the treatment, although bax expression was not affected, which is likely because of the lack of p53 expression in PC-3 cells (Figure 1B). Meanwhile, these *γ*-T3-mediated activation of the proapoptotic proteins as well as the change of bcl-2/Bax ratio were in a dose- and time-dependent manner (Figure 1B), consistent with the effect of *γ*-T3 treatment on inhibition of cell proliferation. In addition, activation of these pro-apoptotic genes after IC_50_ *γ*-T3 treatment (Figure 1C) were only observed in PC-3 and LNCaP cells, but not in PZ-HPV-7, indicating that *γ*-T3 specifically induced apoptosis of androgen-independent prostate cancer cells.

### *γ*-T3 downregulates the pro-survival signalling pathways

**Figure 2:**
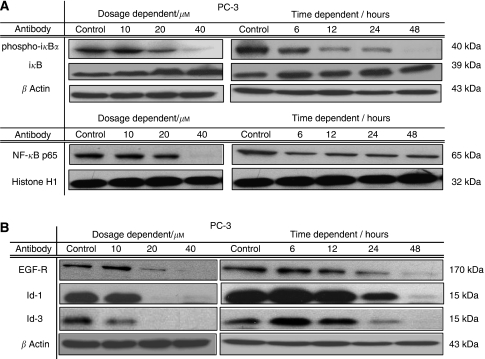
Inactivation of pro-survival pathways by *γ*-T3. (**A**) Effect of *γ*-T3 on the activity of NF-*κ*B pathway was examined by IC_50_ time-dependent and 24-h dose-dependent western blotting (in hours and *μ*M respectively). Note that nuclear translocation of NF-*κ*B p65 and phosphorylated i*κ*B were inhibited by *γ*-T3 treatment. (**B**) Treatment of *γ*-T3 also resulted in downregulation of Id family proteins and EGF-R in PC-3 cells.

Although NF-*κ*B was reported to be constitutively activated in PC-3 (Dhanalakshmi *et al*, 2002), the possibility that *γ*-T3-induced cell apoptosis attributable to the suppression of NF-*κ*B activation was considered. The NF-*κ*B activities of PC-3 treated with *γ*-T3 at either different dosages or at IC_50_ for different periods were measured by examining the translocation of NF-*κ*B subunit p65. As illustrated in Figure 2A, *γ*-T3 treatment suppressed constitutive NF-*κ*B p65 activity in a dose-dependent and time-dependent manner. The effect of *γ*-T3 on NF-*κ*B signalling was further explored by examining the expression of other upstream regulators, such as phospho-i*κ*B*α*/*β* and i*κ*B*α*/*β*. In *γ*-T3-treated PC-3 cells, a time- and dose-dependent decrease in the level of the phosphorylated I*κ*B*α*/*β* were observed (Figure 2A). This is associated with the increase in the level of I*κ*B*α*/*β*, as well as an inhibition of NF-*κ*B p65 nuclear translocation. These results indicate that *γ*-T3 suppressed NF-*κ*B activity through the dephosphorylation and accumulation of I*κ*B*α*/*β*.

Surprisingly, we found that *γ*-T3 treatment also downregulate a number of the key proteins that are involved in the development and progression of prostate cancer. As shown in Figure 2B, EGF-R expression was significantly suppressed to almost an undetectable level by treatment with increasing dosages of *γ*-T3. Similar effect on Id-1 and Id-3 protein level was observed. As EGF-R and Id protein family are essential for cancer cell growth and survival, their downregulation may be associated with the *γ*-T3-induced growth arrest and apoptosis.

### Activation of pro-apoptotic pathway by *γ*-T3 treatment

**Figure 3:**
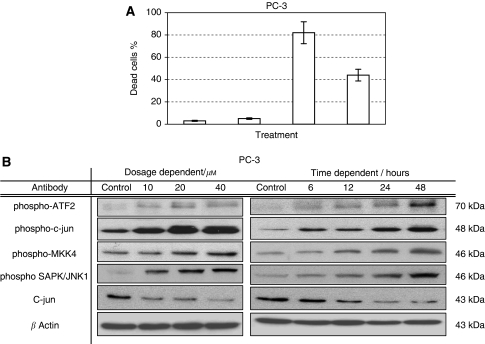
Jun N-terminal kinase (JNK) activation is involved in *γ*-T3-induced apoptosis. (**A**) Cell viability, after incubation with *γ*-T3 and JNK inhibitor (SP600125) for 24-h, was examined by an MTT assay. Note that the addition of JNK inhibitor alleviates the cytotoxicity of *γ*-T3 in PC-3, suggesting that JNK mediates the antiproliferation effect of *γ*-T3. (**B**) JNK activity after 24-h dose-dependent and IC_50_ time-dependent *γ*-T3 treatment (in *μ*M and hours respectively) and was found to be elevated by measuring the phosphorylation levels of MKK4, SAPK/JNK, c-jun and ATF-2. Thus, confirming the involvement of JNK in *γ*-T3 anticancer property.

The c-Jun N-terminal kinase is an evolutionarily conserved serine/threonine protein kinase that is activated by stress and genotoxic agents. JNK phosphorylates the amino terminal of all three Jun transcription factors and ATF-2 members of the AP-1 family. The activated transcription factors modulate gene expression to generate appropriate biological responses, including cell migration and cell death. When PC-3 cells were treated with various doses of *γ*-T3, a dosage- and time-dependent increase in JNK phosphorylation activities were detected (Figure 3B). Meanwhile, phosphorylation of the JNK downstream effectors such as ATF-2 or c-jun were all upregulated by *γ*-T3, supporting that JNK-signalling pathway was activated by the *γ*-T3.

To further confirm the importance of JNK activation in *γ*-T3-induced apoptosis in PCa cells, we investigated whether inactivation of JNK with a specific inhibitor, SP600125, could protect cells from *γ*-T3. As shown in Figure 3A, co-treatment of *γ*-T3 together with 20 *μ*M of SP600125, a dose that was previously determined to inhibit JNK activity in the same cell lines (Uzgare and Isaacs, 2004), decreased the percentage of apoptotic cells compared with that treated with *γ*-T3 alone, confirming that JNK activation may be required for *γ*-T3-induced apoptosis.

### Effect of *γ*-T3 on inhibition of cell invasion

**Figure 4:**
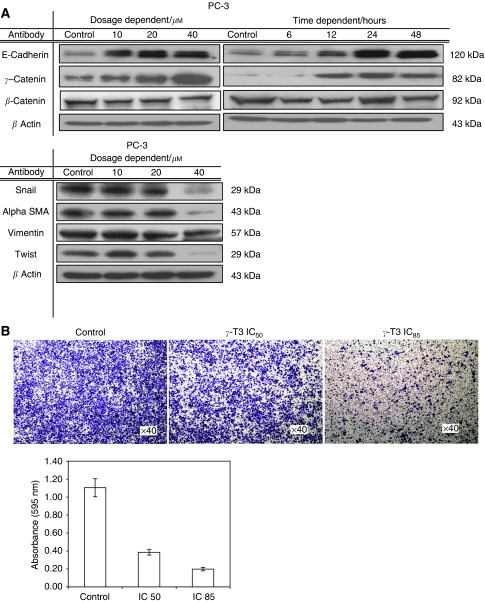
Inhibition of cell invasion by *γ*-T3 treatment. (**A**) 24-h dose-dependent and IC_50_ time-dependent *γ*-T3 treatment induces the expression of epithelial markers (E-cadherin, *γ*-catenin), but suppresses the expression of mesenchymal markers (vimentin, twist and *α*-SMA) and E-cadherin's repressor (snail). (**B**) The invasive androgen-independent PCa cells (PC-3) treated with the indicated dosage of *γ*-T3 was harvested and then plated into the Matrigel-coated (0.5 mg ml^−1^) insert. Cells invaded through the membrane were stained with crystal violet and the images were photographed under a microscope. After being lysed with extraction buffer, intensity at 595 nm was measured.

Although *γ*-T3 has been shown to have antiproliferation effect on many cancers, it is not clear if it affects cancer metastasis. Therefore, we examined whether *γ*-T3 could suppress the invasive ability of the prostate cancer cells. As shown in Figure 4B, using matrigel-invasion assay, we found that *γ*-T3-treated (IC_50_) PC-3 cells for 24 h showed an at least 2.5-time lower invasion capability compared with the untreated control as evidenced by a decrease in the number of cells invaded through the matrigel layer. This inhibitory effect on cell invasion was not the result of cell growth inhibition induced by *γ*-T3 as the number of viable cells added into the invasion chamber were the same. These results indicate that *γ*-T3 is able to inhibit the invasion ability of PCa cells, independent to their cytotoxic effects.

Downregulation of E-cadherin expression is one of the most frequently reported characteristics of metastatic cancers. Restoration of E-cadherin expression in cancer cells leads to the suppression of metastatic ability (Morton *et al*, 1993; Chu *et al*, 2006). In PCa, downregulation of E-cadherin expression is correlated with high-grade tumours and poor prognosis (van Oort *et al*, 2007), indicating their roles in PCa progression. Interestingly, we found that E-cadherin and *γ*-catenin protein expression were upregulated whereas E-cadherin's repressor (snail) (Cano *et al*, 2000) were downregulated after treatment with *γ*-T3 (Figure 4A), although the expression for *β*-catenin remained constant at all treatment dosages and time points. Owing to the deletion of the *α*-catenin in PC-3 cells (Morton *et al*, 1993), there was no expression detected. Separately, the mesenchymal markers (vimentin, *α*-SMA and twist) were all downregulated after treatment with *γ*-T3 for 24 h.

### Effect of *γ*-T3 treatment on Docetaxel-induced apoptosis

**Figure 5:**
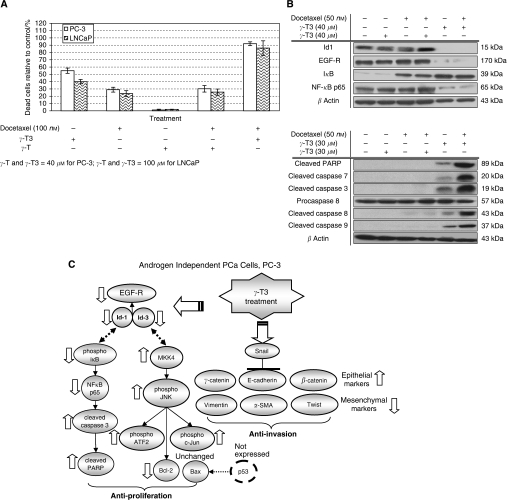
Synergistic effect of *γ*-T3 on Docetaxel-induced apoptosis. (**A**) Effect of Docetaxel and *γ*-T3 co-treatment for 24-h. Cells were incubated with different dosages of *γ*-T3 and 100 nM of Docetaxel for 24 h. Cell viability was examined by MTT assay. The percentage of apoptotic PC-3 and LNCaP cells following co-treatment of Docetaxel and *γ*-T3 was significantly higher than that treated with either agent alone. (**B**) Using western blotting, we further demonstrated that *γ*-T3 co-treatment with Docetaxel for 24-h enhances PC-3 cell apoptosis through activation of pro-apoptotic molecules (cleaved PARP, caspases 3, 7, 8, 9). Additional suppression of proliferation genes were also confirmed for Id-1, EGF-R, i*κ*B, and NF-*κ*B p65. (**C**) Proposed T3 anticancer pathway in PCa cells.

Many of the natural products, such as aged garlic extract (Howard *et al*, 2007) or lupeol (Prasad *et al*, 2008) which are extracted from fruit or plant have been shown to have a chemosensitisation effect. Although it is not clear if *γ*-T3 may affect the sensitivity of cancer cells to chemotherapy, previous study has shown that it did enhance the effectiveness of radiation treatment against prostate tumour (Kumar *et al*, 2006). To test if *γ*-T3 can act synergistically with a chemotherapeutic agent, we have compared the effect of *γ*-T3 alone or in combination with Docetaxel. As shown in Figure 5A, the percentage of apoptotic cells in PC-3 and LNCaP cell lines following co-treatment of Docetaxel with *γ*-T3 for 24 h was significantly higher than that treated with *γ*-T3 or Docetaxel alone. Using western blotting, we further demonstrated that *γ*-T3 co-treatment with Docetaxel enhances cell apoptosis through activation of pro-apoptotic proteins (cleaved PARP, caspases 3, 7, 8, 9) and downregulation of pro-survival proteins (Id-1, EGF-R, i*κ*B and NF-*κ*B p65) (Figure 5B). The level of apoptotic cells is in stark contrast to the *γ*-T co-treatment with Docetaxel. These results suggested that *γ*-T3 and Docetaxel might have a synergistic effect against prostate cancer cells.

## Discussion

Tocotrienol isomers have been earlier shown to inhibit cancer cell proliferation, promote cell cycle arrest, and decrease angiogenesis (reviewed in (Sen *et al*, 2007)). The present report shows that, among eight vitamin-E isomers, *γ*-T3 inhibits PCa cell proliferation through modulation of pro-survival (Id-1, Id-3, EGF-R and NF-*κ*B) and pro-apoptotic (JNK) pathways. Meanwhile, we demonstrated for the first time that *γ*-T3 inhibits cell invasion by restoration of the E-cadherin, *γ*-catenin expression and suppression of mesenchymal markers. Together with the finding that *γ*-T3 enhanced the anticancer effect of Docetaxel, our study provides strong evidences that *γ*-T3 may be developed as a safe and effective anticancer agent for the treatment of prostate cancer.

It is worth noting that published reports (McAnally *et al*, 2003; Conte *et al*, 2004; Vraka *et al*, 2006) indicated earlier that *γ*-T3 isomer suppressed prostate cancer cell growth. In these reports, *γ*-T3's IC_50_ value for PC-3 cells varied between 25–51 *μ*M. The difference between our result and those reported previously could be due to several reasons. Firstly, the IC_50_ values reported earlier were for cells supplemented with *γ*-T3 for 72-h incubation period. Secondly, the *γ*-T3 isomer used in those studies may have different purity than that used in this study (⩾97%). Thirdly, the difference in the proliferation assay used may result in data variation. Although *γ*-T3 treatment caused significant apoptosis in prostate cancer cells, breast cancer cells (Guthrie *et al*, 1997), gastric cancer cells (Sun *et al*, 2008) and human myeloid cells (Ahn *et al*, 2007), several published reports have also indicated that *δ*-T3 is equally potent for inducing apoptosis in other cancer types (He *et al*, 1997; Qureshi *et al*, 2000; Wada *et al*, 2005). For example, HepG2 and B16 melanoma cells treated with *δ*-T3 showed a significant reduction in cell viability with IC_50_ 9.6 and 10 *μ*M, respectively. In our experiments with the androgen-dependent LNCaP cell line, *δ*-T3 is more potent in suppressing the cell proliferation of this cell type among the eight vitamin-E isomers investigated (Table 1A). Taken together, it seems likely that *γ*- and *δ*-T3 may possess tumour-suppressing activities with different cell type specificity and potency.

In this study, we demonstrated that *γ*-T3 suppressed constitutive NF-*κ*B activity through inhibition of i*κ*B kinase activation, leading to apoptosis in PCa cells. This is in agreement with the previous study which showed that *γ*-T3 can interfere with the TNF-induced NF-*κ*B activation pathway in human myeloid KMB-5 cells and several other cancer cell lines (Ahn *et al*, 2007). In addition to their findings, we have demonstrated that *γ*-T3-induced NF-*κ*B inactivation also downregulates the level of bcl-2 in a dosage-dependent and time-dependent fashion. Consequently, this induced apoptosis through activation of caspases 3, 7, 8, 9 and PARP. Consistent with previous results obtained with diverse cell lines differing in p53 status (Mo and Elson, 1999), our results showed that p53 is not required for *γ*-T3-induced apoptosis, as the p53-null cell lines (PC-3 and HL-60 (Mo and Elson, 1999)) are still responsive to *γ*-T3 treatment. It is worth noting that *γ*-T3 was previously demonstrated to abolish NF-*κ*B activation induced by epidermal growth factor (EGF) and other pro-inflammatory cytokines (Ahn *et al*, 2007). Although the molecular mechanism involved was not clear at that time, the authors proposed that *γ*-T3 may act through a common step in the suppression of NF-*κ*B. Our result revealed that downregulation of EGF receptor (EGF-R) was correlated to *γ*-T3-induced NF-*κ*B inactivation (Figure 2B). This finding may explain why *γ*-T3 was able to suppress NF-*κ*B activation by EGF treatment in KBM-5 cells (Ahn *et al*, 2007). Interestingly, the androgen-independent prostate cancer cell line PC-3 was found to be more sensitive to *γ*-T3 treatment than the androgen-dependent LNCaP cells. PC-3 cells were found to have constitutive NF-*κ*B activation and are in general more resistant to chemotherapeutic drug-induced apoptosis than the LNCaP cells. Although the exact reason for this observation is unclear, but based on the fact that non-tumorigenic prostate epithelial cells are highly resistant to *γ*-T3 as well, it is possible that *γ*-T3 may preferentially target the cells with higher malignant phenotype.

We believed that one possible mechanism by which *γ*-T3 could mediate its effects on the NF-*κ*B pathway is through the suppression of Id-1 and EGF-R (Ling *et al*, 2003). We have previously demonstrated that ectopic Id-1 expression in LNCaP cells resulted in increase of NF-*κ*B transactivation activity and nuclear translocation of the p65 and p50 proteins, which was accompanied by upregulation of their downstream effectors Bcl-xL and ICAM-1. In addition, inactivation of Id-1 by its antisense oligo-nucleotide and retroviral construct in DU145 cells resulted in the decrease of nuclear level of p65 and p50 proteins, which was associated with increased sensitivity to TNF-induced apoptosis. Considering these findings, our results strongly suggest that Id gene family may be one of the upstream regulators of NF-*κ*B that is targeted directly by *γ*-T3, and inhibition of NF-*κ*B-signalling pathway may be responsible for *γ*-T3-induced antiproliferation.

In this study, we also showed that c-Jun N-terminal kinase participates in *γ*-T3-induced apoptosis. When PCa cells were treated with *γ*-T3, a series of molecules associated with JNK pathway, such as c-Jun and ATF-2 (Figure 3A), were activated simultaneously. Meanwhile, we demonstrated that treatment of JNK inhibitor (SP600125) protects the PCa cells from *γ*-T3-induced apoptosis (Figure 3A). This further confirms the involvement of the JNK pathway in *γ*-T3-induced apoptosis in PCa cells. It is worth noting that the JNK pathway is also known to be involved in cell apoptosis induced by the chemotherapeutic drug, Docetaxel (Zhang *et al*, 2006). Taking these findings into consideration, we therefore question whether *γ*-T3 possesses synergistic interaction with Docetaxel as a result of activation of the JNK pathway. To this end, we compared the antiproliferation capability of Docetaxel treatment alone, and co-treatment with *γ*-T3. Remarkably, we found that combined treatment of Docetaxel and *γ*-T3, but not *γ*-T, resulted in higher proportion of apoptotic cells (Figure 5A). This finding suggests a possible synergistic role of *γ*-T3 with the chemotherapeutic agent.

In this study, we determined that restoration of E-cadherin and *γ*-catenin expression, together with suppression of snail, *α*-SMA, vimentin and twist, may account for *γ*-T3's inhibitory effect on PCa cell invasion capability. Although the antiproliferation effect of *γ*-T3 has been reported in several cancer types (reviewed in (Sen *et al*, 2007)), our results provide first evidence to suggest that it may also be a potential agent for suppression of cancer invasion. Downregulation of E-cadherin and upregulation of mesenchymal markers (*α*-SMA, vimentin and twist) are some of the most frequently reported phenomena in metastatic cancers (Morton *et al*, 1993). It is suggested that loss of E-cadherin expression is able to promote epithelial–mesenchymal transition (EMT), which plays a key role in the progression of cancer cells to metastatic stage. Although the precise mechanism responsible for E-cadherin inactivation in cancer cells is not clear, alterations at transcriptional level due to its repressor Snail seem to be one of the mechanisms responsible for its decreased expression in several cancer types. In this study, we found that the *γ*-T3-treated PCa cells showed increased E-cadherin expression (Figure 4A), which was associated with reduced Snail protein expression and invasion ability (Figure 4B). Catenins (*α*,*γ*), a family of cytoplasmic cadherin-binding proteins, link E-cadherin to the actin cytoskeleton and are thought to be essential for normal E-cadherin function. In our study, we found that *γ*-T3 only upregulated the expression of E-cadherin and *γ*-catenin, but not *α*-catenin. The expression for *β*-catenin remains static, similar to our previous experiments using garlic derivatives (Chu *et al*, 2006). Although PC-3 cells do not express *α*-catenin, a key molecule for functional E-cadherin expression, *γ*-T3 might restore the function of E-cadherin through other molecules such as vinculin, which has been reported to play a role in the establishment of the E-cadherin-based cell adhesion complex (Hazan *et al*, 1997). Taken together, our results suggest that *γ*-T3 may suppress cancer metastasis through induction of mesenchymal–epithelial transition (MET).

As summarised in Figure 5C, our results demonstrated that *γ*-T3 is a potent and specific inhibitor of PCa cell proliferation and invasion which acts through multiple molecular pathways. As no side effect can be observed after long term intake of natural T3 extract (Patel *et al*, 2006; McAnally *et al*, 2007) (LD_50_⩾2000 mg kg^−1^, data not shown), *γ*-T3 may be used alone or in combination with chemotherapy for treating advanced stage PCa.

## Figures and Tables

**Table 1:** Effect of vitamin-E isomers on prostate cells

**(A)**	**Mean cell viability in percentage (relative to control)**																							
	**Tocopherols (T)**																							
	***α***					***β***					***γ***					***δ***								
**Incubation (hours)**	**24**			**48**		**24**			**48**		**24**			**48**		**24**			**48**					
**Dose (*μ*M)**	**20**	**40**	**80**	**20**	**80**	**20**	**40**	**80**	**20**	**80**	**20**	**40**	**80**	**20**	**80**	**20**	**40**	**80**	**20**	**80**				
LNCaP	106±8	105±5	92±7	102±7	87±5	96±8	92±5	88±5	103±7	88±4	81±7	79±7	67±7	81±4	56±4	103±7	98±5	93±7	84±7	83±5				
PC-3	96±7	101±6	93±5	94±6	91±4	98±9	98±7	95±8	96±9	97±6	92±8	93±5	84±7	88±6	78±6	94±4	92±6	87±5	98±8	85±7				
PZ-HPV7	108±8	102±5	98±5	103±8	104±6	103±8	104±5	99±11	96±8	96±8	99±6	106±5	100±8	97±8	99±7	84±4	95±4	90±7	90±5	94±6				
																								
	**Tocotrienols (T3)**																							
	***α***						***β***						***γ***						***δ***					
**Incubation (hours)**	**24**				**48**		**24**				**48**		**24**				**48**		**24**				**48**	
**Dose (*μ*M)**	**20**	**40**	**80**	**IC_50_**	**20**	**80**	**20**	**40**	**80**	**IC_50_**	**20**	**80**	**20**	**40**	**80**	**IC_50_**	**20**	**80**	**20**	**40**	**80**	**IC_50_**	**20**	**80**
LNCaP	98±6	97±4	90±7	UD	96±7	94±8	93±8	96±7	91±7	UD	97±7	89±7	93±5	89±7	67±4	92	89±8	47±4	96±6	79±6	47±4	75	89±6	42±2
PC-3	105±6	110±5	98±5	UD	99±8	106±7	78±4	61±4	31±3	54	69±4	8±2	63±3	39±2	4±1	32	51±4	6±2	73±8	52±3	10±2	41	61±3	15±2
PZ-HPV7	98±5	97±4	105±6	UD	101±4	101±8	98±6	102±6	91±5	UD	93±8	91±5	94±7	101±3	91±5	UD	88±7	86±4	97±5	99±5	96±8	UD	89±7	86±6
																								
**(B)**	**Mean cell proliferation in percentage (relative to time 0th)**																							
	**Control**						**100 *μ*M *α*-T3**						**IC_50_ *β*-T3**											
**Incubation (hours)**	**0**	**24**	**48**	**72**	**120**	**144**	**0**	**24**	**48**	**72**	**120**	**144**	**0**	**24**	**48**	**72**	**120**	**144**						
LNCaP	100	101±5	121±8	137±6	123±11	157±9	100	95±5	87±6	107±5	113±8	122±10	100	79±8	64±8	44±6	59±7	48±9						
PC-3	100	102±7	106±5	108±9	155±8	174±11	100	102±7	106±9	118±6	136±9	144±9	100	82±8	43±6	48±5	26±4	22±5						
																								
	**IC_50_ *γ*-T3**						**IC_50_ *δ*-T3**																	
**Incubation (hours)**	**0**	**24**	**48**	**72**	**120**	**144**	**0**	**24**	**48**	**72**	**120**	**144**												
LNCaP	100	59±8	49±5	45±5	37±6	32±6	100	57±4	53±4	34±2	15±5	17±3												
PC-3	100	53±6	10±3	8±3	2±2	3±2	100	59±5	18±3	9±2	5±2	3±2												

(A) Cell viability was examined by MTT assay after treatment with different vitamin-E isomers for 24- and 48-h. Note that vitamin-E isomers, particularly tocotrienols, selectively affect the viability of the prostate cancer cells at a different degree, but do not have a significant effect on the non-tumorigenic prostate epithelial cells. PC-3 is more responsive to vitamin-E isomers compared with LNCaP. (B) LNCaP and PC-3 growth rate in the presence of *γ*-T3 at IC_50_. The IC_50_ dose levels correspond to that in Table 1A. For *α*-T3, 100 *μ*M was used. UD indicates undetermined IC_50_.
